# Facilitating access to mental healthcare for patients with long-term conditions in general practice: a qualitative collective case study

**DOI:** 10.1080/02813432.2025.2516497

**Published:** 2025-06-11

**Authors:** Amanda Nikolajew Rasmussen, Viola Burau, Helle Terkildsen Maindal, The Healthy Mind Study Group

**Affiliations:** Department of Public Health, Aarhus University, Aarhus C, Denmark

**Keywords:** Access to healthcare, mental health, long-term conditions, general practice, case study

## Abstract

**Objective:**

Mental health problems among patients with type 2 diabetes (T2D) and ischemic heart disease (IHD) are common, but often undertreated. General practice is a relevant setting for addressing this complex health condition. However, research suggests that this group of patients often experience problems accessing care. This study aimed to explore how access to mental healthcare in general practice for patients with T2D and/or IHD is facilitated by processes of organizing care.

**Design, setting and participants:**

The study was designed as a qualitative collective case study in four general practices in Central Denmark Region participating in The Healthy Mind Study. Data were collected through ethnographic observations of, and qualitative interviews with patients and healthcare professionals.

**Results:**

We found that when healthcare professionals adapted services to specific patient needs, the patients felt welcome and accepted. Furthermore, finding a balance between letting the patient take responsibility while still providing sufficient support led to patients feeling empowered while still feeling cared for. Organizational prioritization of patients in vulnerable situations created circumstances that enabled healthcare professionals to provide better access to care and better treatment outcomes.

**Conclusion:**

Healthcare professionals in general practice must think about their role in facilitating access to mental healthcare. An important part of this is that general practices prioritize patients in vulnerable situations, for example through specialized services, outreach work, structured staff supervision, and extra time. Recruitment of participants for mental healthcare interventions in general practice may benefit from using informal strategies relying on the patient-provider relationship.

## KEY POINTS

- Adapting services to specific patient needs is essential to facilitating access to mental healthcare for patients with long-term conditions. Provider continuity plays an important role in this, and general practice is thus a relevant site for targeting mental health problems among these patients.
- To facilitate the best treatment outcome in mental healthcare for patients with a long-term condition, it is important that healthcare professionals find a balance between facilitating patient empowerment and providing sufficient support.
- Organizational prioritization of patients in vulnerable situations is crucial for creating circumstances that enable healthcare professionals to facilitate access to care. The current study showed how this can be accomplished through specialized services, outreach work, structured staff supervision, and extra recruitment time.

## Background

Patients with long-term conditions, such as type 2 diabetes (T2D) and/or ischemic heart disease (IHD), are more likely to experience mental health problems compared to the general population [1,2]. This co-existence between long-term conditions and mental health is often a vicious circle where loss of meaning and willpower makes it difficult to adhere to treatment, which may lead to worsening of the condition and possibly depression adding to further neglections of treatment [3].

Mental health problems among patients with T2D and/or IHD are often undertreated, partly because this group of patients tends to experience problems in accessing mental healthcare [4–6]. Furthermore, accessing mental healthcare tends to be even worse for patients with low socioeconomic status, long-term unemployment, homelessness, and people from ethnic minorities [6,7].

General practice provides the ongoing care for patients with T2D and IHD in many health systems in the Global North. Healthcare professionals from general practice therefore often have an ongoing, trustful, and informed relationship with these patients [7], which has been highlighted as critical to patients’ access to mental healthcare [8]. However, access problems are also found in general practice [9] and they are even more pronounced when access is related to mental healthcare [10].

When trying to understand unequal access to mental healthcare, patient characteristics are often highlighted, for example patient attitudes, low levels of health literacy, and lack of ability to cover financial health costs [11–14]. Generally, in research and policies concerning social inequalities in health, there has traditionally been a tendency to focus exclusively on individually oriented factors even though comprehensive research has demonstrated the importance of structural, social, and organizational factors [15,16].

The emerging concept of organizational health literacy highlights the importance of understanding the interplay between the ability of patients to access, understand, appraise and apply healthcare services and the ability of healthcare organizations to make it easier for patients to navigate, understand, and use information and services [17]. Access is often mentioned as one of the key dimensions of organizational health literacy in reviews and frameworks, especially for high-risk populations and people in vulnerable situations [17–19]. However, as organizational health literacy is a relatively new concept, there is still a need for theoretical conceptualizations to explain the processes and mechanisms of the concept [18].

Levesque et al. have introduced a theoretical framework for understanding access to healthcare, which, similarly to organizational health literacy, highlights the importance of exploring the interplay between the demand site (patients) and the supply site (the healthcare organization) [20]. Furthermore, factors in this framework influencing access to healthcare are understood not as a static one-time occurrence, but as a process from healthcare needs, perception of needs and desire for care, healthcare seeking, healthcare reaching, healthcare utilization, and consequences of healthcare [20]. Problems with accessing mental healthcare among patients with T2D and IHD are also seen in different aspects of the process of accessing care, including identification of healthcare needs, delayed appointments, communication with healthcare professionals, and treatment outcomes [5,6].

This study aimed to explore how access to mental healthcare in general practice for patients with T2D and/or IHD is facilitated by processes of organizing care. The Healthy Mind Study is used as an example of provision of formal mental healthcare in general practice for these patients. Drawing on Levesque et al. [21], access to healthcare is understood as a process and thus explored both in relation to general access to the general practice, access to the intervention study, and outcome of the intervention study. While the study takes its point of departure in the supply site (the healthcare organization), it draws on the theoretical framework of Levesque et al. [20] in understanding access to healthcare as an interplay between the abilities of the healthcare organization and the patient.

## Materials and methods

### Research design

The study is designed as a qualitative collective case study as outlined by Stake [21]. Case studies enables an exploration of the interrelationship between the case and its context [21]. In the current study, general practices participating in The Healthy Mind Study were included as cases of provision of formal mental healthcare. The study explored how these cases interacted with organizational and patient-related contexts, thereby showing how access to mental healthcare was facilitated by the organization and how the organization responded to different patient circumstances.

In qualitative collective case studies, more than one case is included to explore similarities and differences between different cases [21]. It is therefore important to include cases that show variations relevant for the topic of interest [21]. The Healthy Mind Study was carried out in 12 general practices from Central Denmark Region and four of these were included in the current study. Four cases were considered a relevant number of cases to gain a thick understanding of the different cases while still showing differences among them. As illustrated in Table 1, the cases were included based on the attempt to achieve maximum variation in terms of the size and type of general practice, composition of general practitioners and staff, and geographic location. These criteria were based on information about the practices available for the researchers before initiating data collection. The names of the cases are fictional. Both partnership practices and single practices were included in the study. In Denmark, single practices are owned and driven by one general practitioner, and partnership practices are owned by two or more general practitioners, who share patients, facilities, finances, and staff.

**Table 1.** Characteristics of general practice cases.

	Central health	Forest hill	The Meadow	Heartland
Type of general practice	Partnership	Partnership	Partnership	Single
Number of general practitioners	3	4	3	1
Number of staff	12	14	4	3
Geographic location	Urban	Rural	Rural	Urban

### Setting

The study was carried out in Central Denmark Region between April 2023 and January 2024. In Denmark, healthcare services are tax-financed and free of charge for all Danish citizens. More than 98% of the population is listed with a general practice, and access in the current study is therefore not understood as whether the patient is registered, but whether the patient is in contact with and uses the services in general practices.

General practices in Denmark are self-owned businesses financed through reimbursements from the Danish Regions. These reimbursements are dictated by the collective agreement between the Danish Organi­zation of General Practitioners and the five Danish regions [22]. Nurses and other healthcare professionals are most often employed and perform independent consultations supervised by general practitioners. Patients with T2D and IHD are invited to a chronic care consultation annually, where an overview of the patient’s life situation and illness, treatment, and risk factors for disease progression is obtained. The annual chronic care consultation is often divided into two separate consultations. In the first consultation, relevant paraclinical and lifestyle data are collected and in the second consultation status is assessed, goals are set, and treatment plans are made. According to the collective agreement, general practices can be reimbursed for talk therapy consultations at a 30-minute duration up to seven times during a 12-month period. However, this reimbursement is only provided if the consultations are performed by general practitioners and not nurses or other staff [22].

### The healthy mind study

The current study is a part of a larger research project, The Healthy Mind Study, taking its point of departure in The Healthy Mind Intervention. To examine the intervention, different studies were carried out by The Health Mind Study Group, including a feasibility study [23], a randomized controlled trial (RCT) [24], a process evaluation [25,26], as well as the current qualitative collective case study, which has also been reported in another research article [27].

The Healthy Mind Intervention is a complex intervention offering problem-solving therapy (PST) to patients with T2D and/or IHD in general practice. PST is a cognitive-behavioral treatment focusing on enhancing the patient’s problem-solving skills through empowerment and behavioral activation. Patients are encouraged to take an active role in describing their problems and finding an appropriate solution, and providers act as facilitators [28]. General practice has been considered suitable for performing PST because the treatment is based on short consultations and requires little training [28,29] The method has been reported as relevant for patients with psychological problems secondary to medical conditions [28]. Research has suggested that PST can improve mental well-being and glycemic control for patients with T2D [30,31], while the evidence base for the effect on patients with IHD is limited. The Healthy Mind Study was initiated to explore this effect.

In the Healthy Mind Study, 12 general practices from Central Denmark Region were included to explore the effect and implementation of The Healthy Mind Intervention. Healthcare professionals from the practices attended a two-day course on the PST method. Patients were recruited for the intervention as a part of their annual chronic care consultation. Based on the WHO-5 Well-being Index [32], patients were screened and invited to participate in the study if they were categorized as having poor mental well-being. The WHO-5 Well-being Index is a widely used and validated screening tool based on five questions. The questions concern whether the respondent during the last two weeks has felt (1) cheerful and in good spirits, (2) calm and relaxed, (3) active and vigorous, (4) that they woke up feeling fresh and rested, (5) that their daily life has been filled with things that interest them. Patients were excluded from the study if they suffered from severe mental illness (psychosis, suicidal mental state or dementia) or if they were unable to understand, read or write Danish. The patients participating in the intervention study had a mean age of 62.6 and were 63% male and 37% female. The participating patients were offered up to seven 30-minute PST consultations. The idea was to perform the consultations weekly, but this was adapted according to patient preferences and possibilities. Worksheets were used in the consultations as a pedagogical tool to guide the patient through five steps consisting of (1) listing problems and selecting one to work with (2), defining the selected problem (3), generating possible solutions (4), analyzing strengths and weaknesses for each solution and choosing one (5), implementing the chosen solution [24]. The practices were reimbursed by the project team for the time they spent on both screening and consultations. During participation in the intervention study, contrary to normal practice, the general practices were reimbursed for both general practitioners and staff members performing the consultations. In most practices, the PST consultations were performed by staff members, especially nurses.

The RCT study was carried out simultaneously with the intervention, and is therefore closely intertwined with, and difficult to separate from, the intervention. For example, when the participants were included in the intervention, they were also included in the RCT study, and the RCT study thus determined how the participants were recruited. Drawing on Levesque et al. [20], the current study understands access as a process involving both general access to the general practices, access to the intervention study, and outcome of the intervention study. The study therefore explores both recruitment for the RCT study and the intervention. In the following, to avoid confusion, ‘The Healthy Mind Study’ refers to the RCT study and the intervention, as the combination of these two parts are the focal point of exploration in the study.

**Table 2.** Overview of data.

	Central health	Forrest hill	The meadow	Heartland
Interviews with clinic owners	2	2	2	2
Focus group interviews with staff members	2	2	2	2
Interviews with patients	2	2	2	2
Days of etnographic observations	3	3	3	3

### Data collection

In qualitative collective case studies, multiple sources of data are often deployed to provide different perspectives and thereby a holistic account of the case and the context [21] In this study, 12 days of ethnographic observations [33], 8 individual semi-structured interviews [34] with clinic owners, 8 focus group interviews [35] with staff members, and 8 individual semi-structured interviews [34] with patients were used to capture different perspectives on the cases and the contexts (Table 2). The participants were sampled to create maximum variation in terms of age, gender, years of employment in the practices, illness conditions, years of living with the condition, and number of PST-consultations. By conducting separate interviews with clinic owners, staff members, and patients, each group was afforded the opportunity to express perspectives and concerns they might have been unwilling to share in the presence of the other parties.

Three days of ethnographic observations [33] were conducted in each case. Fieldnotes were noted down in handwriting during the observations. To explore the supply context, and thereby the unique culture and structure of the practices, the ethnographic observations focused on the everyday life of the practice and included observations of different consultations, staff meetings, lunch breaks, and secretary work. Furthermore, to explore access to the intervention, observations of screening and recruitment of participants to The Healthy Mind Study were conducted in all practices. Observations of PST consultations were also planned in all practices. However, staff in all practices but one was not comfortable with having a third person (the observer) present at PST consultations. Consequently, only one PST observation was made in one practice.

Individual semi-structured interviews [34] were conducted with a clinic owner in each case. The interviews were guided by an interview guide (Supplementary material) but open to unexpected relevant turns in the conversation [34] and transcribed by the first author. These interviews generally made it possible to gain a sense of the ideas and visions for the general access to the general practice. Furthermore, as the clinic owners were often the initiators and the project managers of the intervention, they also provided perspectives on their experience of the organization of the intervention. To explore how the process of facilitating access was affected by the healthcare professionals’ experience with the PST-method, and the overall implementation process, two interviews were conducted for each case, one at the beginning, and one at the end of the intervention period. This also made it possible to elaborate on topics in the second interview that were not sufficiently discussed in the first interview.

Focus group interviews [35] were conducted with the staff participating in The Healthy Mind Study in all practices. The participants in these focus group interviews were nurses, except for one participant who was a general practitioner who was not a clinic owner. For the same reasons a with the one-person interviews with the clinic owners, two focus group interviews were conducted at the beginning and the end of the intervention period. The focus group interviews provided insights into factors inhibiting and promoting access to the intervention, seen from the organizational perspective. Focus group interviews enable insights into social group dynamics and norms [35] and thereby made it possible to explore the organizational workplace culture and context. The focus group interviews were semi-structured [34] and guided by a list of themes (Supplemental material) and transcribed by the first author. However, in focus group interviews, the questions asked by the researcher are more open-ended and try to foster discussions among the participants [36].

As a part of the last focus group interview in each practice, two vignettes (Supplementary material) were included describing fictional examples of patients participating in the intervention. The vignettes made it possible to explore complex public health issues by placing statements and experiences in a concrete context [37]. As it was not possible to observe PST consultations in all practices, the vignettes enabled concrete and contextual insights into these consultations. The two vignettes represented patients with a relatively high and low level of health literacy, vulnerability, and social support. By letting the healthcare professionals explain how they responded to these different patients, it was possible to explore how they would provide the best possible access for them, and how this perhaps differed according to the different patient characteristics. The thoughts and theoretical notions behind the vignettes were not explained to the healthcare professionals to avoid reproduction of these concepts. The vignettes were developed by the authors, who have substantial experience and knowledge of health literacy, patients in vulnerable situations, and access to complex interventions as well as a psychologist specialized in the PST method.

Individual semi-structured interviews [34] were conducted with two patients participating in The Healthy Mind Study from each case. These interviews were guided by an interview guide (Supplemental material) but open to unexpected relevant topics [34] and were transcribed by the first author. The interviews enabled insights into the patient’s perspective on their ability to access care in general practice and use of services, as well as their experience of the general practice and the healthcare professionals’ facilitation of access. The patients differed in terms of number of PST-consultations, which made it possible to explore patients that had dropped out after only one consultation as well as patients that had completed up to five consultations. However, unfortunately it was not possible to include patients that declined participation in the intervention study.

### Data analysis

The data analysis followed the three phases of data analysis in qualitative collective case studies as described by Stake, including categorical aggregation, establishment of patterns, and naturalistic generalizations [21]. First, the transcribed interviews and ethnographic field notes were coded and categorically aggregated using NVivo software. Drawing on Levesque et al. [20], an in-depth understanding of each of the cases was obtained by identifying factors on demand and supply aspects affecting access to the general practice, access to the intervention study, and outcome of the intervention study. Second, patterns across the four cases were identified by comparing the categorical aggregation within the cases and identifying themes across the cases. In this phase, three themes were developed, which are presented in the Results section. Naturalistic generalizations are conclusions made through personal engagement in life’s affairs. To validate these generalizations, similar experiences are included through theory and existing research. This is illustrated in the Discussion section, where the main findings are summarized and discussed in relation to existing literature. The theory of Levesque et al. [20] has been used as a theoretical framework underpinning the focus of the study and as a means to validate the naturalistic generalizations together with other theories and research.

### Ethics

The Central Denmark Region Committee on Health Research Ethics approved the study with the study ID: 1–10-72–68-22VEK.

Informed consent was obtained in different ways. Before conducting ethnographic observations in the practices, the first author introduced herself and the qualitative collective case study for all clinic owners and staff members at a staff meeting. This was done to make sure all professionals understood what the purpose of the study was and specifically what the ethnographic observations entailed. The healthcare professionals were told that they were welcome to contact the researcher if they did not want to be included in the observations. Furthermore, prior to each interview, all participants were asked to fill out an informed consent contract.

The researchers were aware that the patients participating in the study were potentially in a vulnerable situation of living with a long-term condition and experiencing mental health problems. Moreover, by being involved in the intervention, the patients were already burdened by different tasks, including filling out questionnaires and showing up in the practices several times. When being recruited for this qualitative collective case study, we therefore stressed that the patients could withdraw anytime. Furthermore, the interviews were carried out close to the patients’ homes to minimize their time and energy spent on transportation.

The healthcare professionals involved in The Healthy Mind Intervention were also already under extended pressure as they were asked to perform several new tasks, often without having extra time to do this. Furthermore, conducting mental health consultations was a completely new, and rather sensitive task, especially for the nurses in most practices. The researchers therefore made sure not to place additional pressure on the healthcare professionals, and respect their boundaries, when they for example were not comfortable with the researcher participating in the PST-consultations.

## Results

Based on the first two phases of the analysis, categorical aggregation and establishment of patterns, three themes were identified as important for facilitating access to mental healthcare in the four participating general practices: Adapting services to patient needs; Balancing patient empowerment and provider support; Organizational prioritization of patients in vulnerable situations.

### Adapting services to patient needs

To facilitate access to mental healthcare in the general practices, it seemed essential that the practices adapted their services and approaches to the individual patient. Through this, they could accommodate different patient needs and make sure all patients were able to access the physical surroundings of the practice, understand the written and oral communication, and felt that their specific situation was taken into account by the healthcare professionals.

Adapting services to the needs of the patients were both relevant in terms of the general access to, and outcome of the everyday treatment in the practices and in the access to, and outcome of, The Healthy Mind Study.

An example of patient adaptation in the general access to, and outcome of treatment in the practices, was that the practices used categorization in the medical record system of patients in vulnerable or complex situations. This made it possible for all staff to be aware of special needs among this group of patients and accommodate these. The practices defined ‘vulnerability’ and ‘complexity’ differently, but it was often related to multimorbidity, mental health problems, and/or social problems. These patients were often assigned to specific providers to ensure provider continuity and thereby a close and trustful relationship. The patients generally explained that they appreciated when their vulnerabilities were acknowledged and accounted for by the healthcare professionals, because it made them feel welcome and accepted. This is exemplified in the following citation:

I told her [nurse, red.]: “Listen, at the moment I am dealing with some challenges.” And then she noted it on the front page of my record. And that was just… that was kind of like an acknowledgement of well… that I was also welcome, even with all the things that… with all the things that I bring with me. (Patient, Heartland).

Similarly, adapting services to specific patient needs was also relevant for the recruitment of participants in The Healthy Mind Study. The healthcare professionals explained that for the patients to feel that the intervention was relevant for them, the professionals had to adapt their communication tone, level of information, and argument for participating, to the specific patient’s situation, mood, needs, mental literacy level, and personality. This was significantly easier if the professionals knew the patients well.

However, the RCT study design in The Healthy Mind Study meant that the recruitment procedure had to be rather standardized and formal, including signing of documents, and screening through the WHO-5 Well-being Index. This seemed to create some limitations in terms of adapting the recruitment to the individual patient. For example, in The Meadow, before participating in the Healthy Mind Study, the practice was already assessing the mental well-being of their patients as a part of their annual chronic care consultation and offering eligible patients mental health consultations. However, in the Healthy Mind Study, the practice failed to recruit as many patients as they used to. One of the general practitioners explained this:

The recruiting is a bit more… not as informal as we are used to. And not as… we will usually say:” I can feel that you are different.” We have known these patients for a long time so if they start looking a bit sad, right, we will normally carefully push them in the direction of: “Maybe you should…?” or “Are you sure that…?” In the randomized controlled trial, when you must tick boxes and interpret results it is a bit more clinical and not as relational. (General practitioner, The Meadow).

The formalized recruitment procedure in the study made it difficult to base the recruitment on the healthcare professionals’ relationship with and knowledge of the patient. Instead of looking at the individual patient and deciding what course of action to take, based on their knowledge of the patient, the clinic owner explained that they had to base their solutions for the patient on their scores in the WHO-15 Well-being Index, which did not always show the same results as they could observe in the patients. Furthermore, the clinic owner explained that the formal nature of the recruitment in the intervention did generally not fit the temper of the patients in The Meadow, who tended to need a more informal and relaxed attitude towards mental health issues. This illustrates the importance of being able to adapt the recruitment strategy to the individual patient.

While there were different circumstances, priorities, and possibilities in respectively the general access to, and outcome of the treatment in the practices and in The Healthy Mind Study, the findings showed that in both situations it seemed crucial to adapt the services to the needs of the patients in order for the patients to feel welcomed and a relevance of the treatment.

### Balancing patient empowerment and provider support

Finding a balance between helping the patient achieve a sense of empowerment while still providing sufficient support appeared to be an important task for healthcare professionals to facilitate the best outcome of the treatment.

Several patients and healthcare professionals in the study explained that when the patients managed to take responsibility of their situation and gain ownership of their problems, they felt empowered and full of renewed energy. It was also an essential part of the PST approach is to enhance the patients’ problem-solving skills by letting them formulate their problems and find appropriate solutions to them.

An example of empowerment achieved through the course of PST consultations is illustrated by a patient in Forrest Hill. The nurse who had performed the PST consultations with this patient explained that she had experienced a dramatic change in the patient, both mentally and physically. This change was developed during the course of the PST-consultations, but the nurse could already tell a difference after the first consultation:

“He is like… he has many, many problems and he is often very apathetic. I usually have to pull things out of him. And he suffers from a condition that makes it difficult for him to sit right up. But then, when I picked him up in the waiting room for his second consultation, he was like a completely different person. He sat there with his book, and he had prepared everything, and he was ready and happy to talk. And he actually straightened his shoulders which he normally can’t. He got responsibility. And he took control of his life.” (Nurse, Forrest Hill).

This patient was also interviewed by the researchers, and he confirmed how the PST consultations had made him feel empowered, in control, and capable of solving his problems.

However, several patients also explained that they sometimes needed the healthcare professionals to take on more responsibility for the consultation, because they did not have the energy or ability to do it themselves. They explained that it was important that they felt support from the healthcare providers in the PST consultations and not felt alone with their problems. This is seen in the following citation, where a patient explains what she missed in the PST consultations:

"It’s not always that you have strength and energy. And when you don’t have strength and energy, your brain doesn’t function as well. So, it’s not always that you can solve your problems on your own. And it would have been nice if she [the nurse, red.] had said, ‘Listen, I can tell that you are struggling to find a solution. I think it would be good for you to do this and that.’ That’s probably what I needed most at that moment." (Patient, Central Health).

Based on their experiences from recruitment in the annual chronic care consultations and their engagement with the patients in the PST-consultations, the healthcare professionals explained that some patients seemed to be in a highly complicated or vulnerable situation to have the energy needed to take on the necessary responsibility of the PST consultation. This group of patients often neglected participation or withdrew from the intervention study before or shortly after the first consultation. Some of the patients who only attended one or two consultations also explained that one of the reasons for dropping out of the intervention study was that they did not feel enough support from the healthcare professionals.

It thus seemed as though the healthcare professionals had to find a balance between providing enough support for the patient while still letting them take control and be empowered. The following is an example of a patient who experienced that the provider managed to find this balance:

“I think it has been a perfect combination of… She [the nurse, red.] could really sense when I needed to just sit and think for two minutes and reflect on my own, or when it simply became overwhelming or too… perhaps too difficult. I think her sense of when I needed to either bounce ideas off her or needed guidance or… Her sense of that has really been good.” (Patient, Heartland).

The healthcare professionals also explained that they needed to distinguish between how much support different patients needed. Here, the importance of patient adaptation, also described in the previous section, seemed especially crucial. The providers argued that it was important to know the patients and thereby be able to tune into their specific needs in specific situations. This was especially seen when the healthcare professionals were presented with the two fictional vignettes of patients participating in the intervention with different resources and capabilities. Here, all professionals argued that they would adapt their approach differently to these two patients, being more or less passive. For example, one of the vignettes described a patient who had trouble putting his experiences and emotions into words. In this case, the professionals argued that he needed more support in finding those words compared to the other vignette, describing a more resourceful patient.

### Organizational prioritization of patients in vulnerable situations

Management investment in The Healthy Mind Study and organizational prioritization of patients with T2D and IHD in vulnerable situations generally seemed important for facilitating access to mental health care in general practice.

In most of the practices, the healthcare professionals were aware of a group of patients that never or rarely showed up in the practices despite their potential need for healthcare. The clinic owners explained that this group of patients was not a major priority and that they could do more to provide better access for them.

However, in Forrest Hill, an important part of the overall vision was to provide specialized services to patients in vulnerable situations. This practice was organized to allow the general practitioners to spend more time on tasks outside the usual scope of general practice. For example, some of the general practitioners performed structured outreach work, where they regularly visited social drop-in centers and socially disadvantaged areas in the community and provided on-location informal healthcare to meet the patients in safe surroundings.

When trying to explain the vision of the practice, one of the clinic owners referred to a well-known Danish health researcher, Morten Sodemann, who used the picture of a cabinet too filled with plates to illustrate why it is difficult and time-consuming but essential to prioritize patients in vulnerable situations and their mental health [38]:

It’s a cabinet with a glass door. It’s a rather nice metaphor, really. Everyone sees what’s inside the cabinet, but they all just walk past it, thinking: I don’t want to be the one opening it, because then everything will fall out. So, if you want to run a healthcare system solely based on time efficiency, then you should avoid opening the cabinet, right? And you should avoid asking about people’s mental health. You should just ensure that their diabetes treatment is managed. But if you want to do something good, then you must dare to open the cabinet. But it is time-consuming, isn’t it? (Clinic owner, Forrest Hill).

In the Healthy Mind Study, managerial investment in the project meant that different initiatives were implemented which made it easier for the healthcare professionals to provide better access to the intervention and ensure better outcomes of the PST consultations.

An example of this was seen in the case of Central Health. Here, structured supervision consultations were organized for the healthcare professionals who were a part of The Healthy Mind Study because the clinic owners experienced that the healthcare professionals needed extra support. As illustrated in the following citation, the healthcare professionals explained that this was essential because it was a completely new task to be performing mental health consultations for most of them:

I couldn’t have done it without these supervision sessions. I believe it is absolutely necessary that the therapist feels secure doing this completely new thing, so that the patients also feel like they’re in good hands (Nurse, Central Health).

Furthermore, the clinic owners in Central Health decided to prolong the annual chronic care consultations from 15 to 30 min while the practice participated in The Healthy Mind Study. The healthcare professionals in the other practices, where the annual chronic care consultations were still 15 min, explained that the agenda of these consultations was already packed, and that they struggled to squeeze in another task without extra time. This sometimes meant that the healthcare professionals did not prioritize recruitment for the study. However, at Central Health, the healthcare professionals explained that they had time to tune in to the specific patient and adapt the recruitment strategy to their needs. Central Health also succeeded in recruiting most participants relative to the size of the practice.

## Discussion

### Main findings

The findings of the study showed that adapting services to specific patient needs made the patients feel welcome and accepted. The ability of healthcare professionals to find a balance between letting the patient take responsibility while still providing sufficient support left the patient with a feeling of being empowered while still feeling supported and cared for. Organizational prioritization of patients in vulnerable situations enabled the healthcare professionals to provide better access to and outcomes of the treatment. In the following, these naturalistic generalizations will be validated and discussed in relation to existing research and theory.

### The importance of patient adaptation and provider continuity

The study showed that adapting services to the individual patient’s needs was an important part of facilitating access to mental healthcare for the patients with T2D and/or IHD. By exploring access as a process, the study demonstrated how patient adaptation is important both in terms of general access to the practices as well as access to and outcomes of the intervention study. Furthermore, the study indicated that adaptation was closely related to provider-continuity as it was easier for the healthcare professionals to tailor the treatment to the individual patient if they had a thorough knowledge and understanding of the patient. Several studies have similarly shown that continuity of care is especially important for patients with mental health problems and comorbid long-term conditions, as provider continuity may reduce use of psychiatric services, symptom severity, suicide attempts, and improve quality of life and social functioning [39–42]. General practice has been highlighted as an important provider of continuity of care [43], which makes it a relevant site for targeting mental health issues among patients with long-term conditions [1,2,44,45]. Different initiatives have been carried out in general practice to target these issues, including interventions to support general practitioners, telemedicine interventions, and therapeutic interventions [46]. The effectiveness of these interventions is often contingent upon healthcare professionals’ in-depth knowledge of their patients, as well as the establishment of a trusting patient-provider relationship, which, in turn, is facilitated by provider continuity [44].

However, the current study also showed that due to the design of the RCT, it was difficult for some of the healthcare professionals to base recruitment on their relationship with and knowledge of the patient. This partly explained why a lower number of participants were included in the study than expected. Research has demonstrated that patients with mental health problems and long-term conditions are generally difficult to reach [4–6]. Furthermore, problems with recruiting patients in vulnerable situations for clinical trials have also been identified in several research projects [47]. For example, The SOFIA Study, a similar mental health intervention study was carried out in general practices in Denmark, and also showed difficulties in recruiting this complex patient group [48]. Here, being familiar with the patient was also highlighted by the healthcare professionals as an important determinant of successful recruitment. RCTs is the gold standard to investigate the effect of a health intervention. However, the formalized recruitment implied in the RCT design may not be the most appropriate in interventions targeting people with mental health problems in general practice, as shown in this study.

### The joint responsibility of patients and providers

The healthcare professionals in the study argued that the reason why some participants declined participation or never showed up or only completed one PST consultation was that they seemed to be in too complicated or vulnerable situations to take on the necessary responsibility in the PST consultation. This could be explained through the concept of individual health literacy, which explains healthcare access inequalities through lacking resources, abilities, and knowledge among individual patients [49]. However, the current study also showed the important role of healthcare professionals in adapting their treatment to the individual patient and providing sufficient support. Similarly, a second wave of health literacy research has shifted their focus from solely emphasizing individual patient characteristics towards also focusing on how the healthcare professionals adapt their treatment to these characteristics [50]. An important part of this wave is the notion of organizational health literacy, which highlights the role of the organization in responding to different health literacy levels [17]. Furthermore, the framework of Levesque et al. shares a similar focus on the interplay between demand and supply site but with a specific focus on access [20].

The study also showed that organizational prioritization of patients in vulnerable situations and management investment in the intervention seemed to be important for facilitating access to and outcomes of treatment. In most reviews and frameworks of organizational health literacy, leadership prioritization, procedures and policies, staff training, sufficient time, and organizational culture of innovation and change are highlighted as important prerequisites for being a health literacy responsive organization [17,18,51–54]. However, the concept of organizational health literacy needs further theoretical and empirical elaboration and exemplification to show how this can be accomplished in practice [20], which in the current study was illustrated in terms of mental healthcare in general practice. Providing specialized services, performing outreach work, facilitating structured supervision for staff members that are not used to carry out mental health consultations, and extra time to recruitment were activities initiated by the management in the current study, which made it easier for the healthcare professionals to provide better access to mental health care.

### Strengths and limitations

A core strength of the study is the qualitative collective case study design, which enabled accumulation of comprehensive and multifaceted data material. By exploring access both from the supply site (healthcare professionals) and the demand site (patients), the study showed the joint responsibility of the two parties in creating access. Furthermore, by conceptualizing access as a process and thereby exploring general access to the practices as well as access to and outcome of the intervention study, the study was able to show the difference between the formalized nature of the intervention study and the informal character of normal practice.

The study also has limitations. As the study explores facilitation of access, it is a limitation that none of the patients who did not access the intervention study were included as they could have provided relevant insights about what prevented them from gaining access. However, it was not possible to contact patients who declined participation in the intervention study because they had not given consent to provide their contact information to the intervention team. It would perhaps also have been unethical to make contact to these patients who, for different reasons, did not want to engage in a research project at the time. Nevertheless, the study did include patients who withdrew from the intervention study after one or two consultations and these patients may provide similar insights about access and outcome complications.

## Conclusion and implications for practice

The study showed how access to mental healthcare in general practice for patients with T2D and/or IHD can be facilitated through processes of organizing care. First, adapting services to the individual patient’s needs appeared important and seemed closely related to provider-continuity. Second, for patients in particularly vulnerable situations, such as patients with long-term conditions and mental health problems, it is critical to manage the balance between patient empowerment and provider support. Last, organizational prioritization of patients in vulnerable situations seems to be important for creating the best circumstances that enable healthcare professionals to facilitate access to care.

The study indicated that providing mental healthcare with the use of the PST method in general practice for patients with T2D and or/IHD seemed relevant, both for patients and healthcare professionals. However, the study also showed that even in an intervention setup where resources were allocated for the practices to be able to offer this treatment, it was still difficult for the healthcare professionals to be able to facilitate access to the treatment.

For healthcare professionals in general practice to be able to facilitate access to mental healthcare they must think about their role in facilitating access to mental healthcare and especially try to adapt their treatment to the individual patient and find a balance between promoting patient empowerment while still providing sufficient support. An important part of this is that patients in vulnerable situations are prioritized by the organization, and that initiatives, such as specialized services, outreach work, structured staff supervision, and extra recruitment time are implemented. Furthermore, if aspects of the Healthy Mind Intervention or other mental health interventions are going to be implemented into the daily work of general practices, it may be necessary to rethink recruitment of participants and possibly base it on the relationship between the patient and the professional, instead of applying formalized recruitment strategies such as screening through the WHO-5 Well-being index.

## Supplementary Material

Supplementary material.docx
