# Supplementary material for: Facilitating access to mental healthcare for patients with long-term conditions in general practice: a qualitative collective case study
Source: Scand J Prim Health Care. 2025 Jun 11;44(1):1–13. doi: 10.1080/02813432.2025.2516497 (PMC12918328; doi:10.1080/02813432.2025.2516497)
Supplement: Supplementary material.docx [file IPRI_A_2516497_SM6075.docx]

**Interview Guide, Focus Group Interview with Staff**

**Introduction to the interview**

The purpose of my part of the study is to identify key considerations for scaling the problem-solving method to various practice types and patient groups.

This is a confidential space. I will not attribute specific comments to individuals. Please feel free to be honest. This is an opportunity for you to share your opinions.

Today, we will cover three themes. First, I’d like to hear about your experiences working in this practice. Then, we’ll discuss your experiences with *The Healthy Mind Study*. Lastly, we’ll talk about different types of patients.

We’ll begin with a round where everyone gets a chance to speak without interruption. After that, it will be a more informal conversation where you are encouraged to comment on each other’s points, and I’ll ask follow-up questions.

**The organization**

- *Introductory round***:** Please introduce yourselves by stating your name, how long you’ve worked here, and your experiences working here—what are the positives and challenges? How does it compare to other places you’ve worked?
- How do you perceive collaboration between the different professional groups?
- How do you experience the management?
- How would you describe the work environment?

**The Healthy Mind Study**

- How do you think the patient screening is going? What challenges do you experience?
- What do you think of the method? Does it make sense to you? Are you confident in using it?
- What do you think about taking on a more passive role as a practitioner?
- How does the method differ from how you usually interact with patients? Has it changed your role?
- For those of you who have conducted sessions, what has been your experience?
- How does the leadership support you regarding the sessions?
- What do you think is needed to make it work better for the remainder of the period?

**Different Types of Patients**

- How well do you think this practice manages to engage with vulnerable patients? Is there a culture around this? Official procedures?
- Do you consciously adapt your communication or behavior towards different patients in specific ways?
- Do you do anything to build patients’ skills or capacities?
- Have you noticed any differences in how it feels to screen different types of patients?
- How well does the problem-solving method fit with different patient types? E.g., socioeconomic status, social support?
- What do patients need to be capable of to benefit from the sessions? Can you teach them these skills?
- Are there specific patient groups for whom the method works particularly well or poorly?

**Interview Guide, Individual Interview with Clinic Owner**

**Introduction to the Interview**

The purpose of my part of the study is to explore key considerations for scaling the problem-solving method—how it fits into different types of practices and for various patient groups.

We will cover three themes. First, I’d like to hear a bit about your practice in general. Then, I’d like to discuss The Healthy Mind Study and how you feel the implementation of the problem-solving method has worked in your practice. Lastly, I’d like to talk about different types of patients.

Your participation is anonymous, and this conversation is confidential. In the reporting of the project, no information will be shared that could directly identify you.

**The organization**

- Tell me about yourself—why did you choose to work in general practice?
- Tell me about your practice—how was it established, and what is the vision and idea behind it?
- In what ways does your practice differ from others?
- How is your practice structured? What are the roles of the doctors and the staff?
- When hiring, is there anything specific you look for?
- How do you experience collaboration with your staff?
- How do you experience collaboration with the other doctors?
- How would you describe the culture in your practice?
- How do you experience being a leader?

**The Healthy Mind Study**

- How—and why—did your practice become part of The Healthy Mind Study?
- How have you approached the study? Who conducts the sessions, and how do you screen patients?
- How do you feel the patient screening process is going?
- What are your thoughts on the problem-solving method? Does it make sense? Does it resemble the type of consultations you already conduct?
- How have staff reacted to conducting these problem-solving sessions?
- Do you do anything to support the staff in relation to these sessions?
- What challenges have you encountered during The Healthy Mind Study?
- What do you think is needed to make it work better for the remainder of the period?

**Different Types of Patients**

- Can you describe your patient demographic? Do you have a higher proportion of a particular type of patient?
- Do you have specific strategies for engaging with resource-poor patients? If so, what are they?
- Do you take any specific measures to ensure that patients facing challenges (physical, social, or mental) in accessing your practice can get care? If so, what?
- Do you adapt your verbal and written communication for different types of patients?
- Is staff supported and trained to handle vulnerable patients in specific ways?
- Is the concept of health literacy something you work with?
- How do you think the problem-solving method suits different types of patients?
- Are there patient groups for whom the method works particularly well or poorly?
- Do you take any steps to adapt the problem-solving consultations for different types of patients?

**Interview Guide, Individual Interview with Patient**

The purpose of this interview is to explore what works and what doesn’t with the problem-solving therapy—helping to identify key considerations for its broader implementation and assess how it fits into different medical practices and for various types of patients.

This is a confidential space. Your name will not appear anywhere, and I won’t share what you’ve said with your medical practice or include it in our reporting in a way that could identify you. Please feel free to be completely honest. May I record our conversation?

Today, we will cover three themes. First, I’d like to learn a little about you. Then, I’d like to hear about your experience with your general practice. Finally, we’ll discuss your experience with the problem solving therapy consultations.

**About You**

- Tell me briefly about yourself—family, education, work.
- Do you have type 2 diabetes or heart disease?
- When you joined this research project, you filled out a short questionnaire that indicated relatively low mental well-being—how do you perceive that?

**Your General Practice**

- How long have you had been a patient in this general practice?
- Why did you choose this particular practice?
- How does this practice compare to the one you had before?
- Do you have a good relationship with one or more of the staff? Any positive experiences?
- Are there any staff members you don’t like as much? Any negative experiences?
- Have you ever found it difficult to understand what the doctors or nurses are saying to you?

**Problem-Solving Therapy**

- As part of your annual check-up, you were asked some questions about your mental well-being and then invited to join the project—how did you experience that?
- Were you hesitant about participating? What made you decide to join?
- Have you found it easy to understand what the method used in the sessions is about?
- Who are you having the sessions with? What is your relationship with this person?
- How many sessions have you had? What has your experience been like?
- Do you feel that the doctor/nurse takes on a different role in these sessions compared to usual?
- How do you feel about being the one who identifies your problems and solutions? Does it resemble the way you typically receive help from your doctor/nurse?
- Have you ever had this type of session with your general practitioner before?
- Do you think these sessions will help you?
- What do you think could improve the sessions?

**Vignettes**

**Vignette 1: Hans**

Hans is 71 years old. He is a retired carpenter, divorced, and lives alone. He has two adult children but has lost contact with one of them. Recently, Hans has noticed that he feels more fatigued and sadder.

During his annual chronic care consultation for his type 2 diabetes, the nurse tells him about a research project. She has a stack of papers filled with text, which reminds Hans of a school assignment and brings back bad memories from his school days. Hans tells the nurse, “I’m not sure this is for me. I don’t think I can manage it.” The nurse reassures him that she will help him through it and believes it could benefit him. Hans agrees to participate.

During the first session, Hans is given a book, and they go through the first two steps together. Hans is asked to identify the problems he is currently experiencing and choose one to work on. Hans decides to focus on his sadness. The nurse encourages him to explore this further and asks, “Why do you think you feel sad?” Hans finds this difficult to answer. The nurse then asks when he particularly notices feeling sad. Hans replies that it’s in the evenings when he’s sitting on the sofa. “I guess I feel a bit lonely,” he says. Together, they decide to focus on tackling his loneliness.

At the next session, they move on to the next step, which involves discussing possible solutions to Hans’ problem. The nurse asks about Hans’ social relationships, and he explains that they often end in conflict. The nurse asks, “Can you identify a pattern in what goes wrong?” Hans struggles to see any patterns. He also doesn’t fully understand why they are discussing this and asks, “Aren’t we supposed to find solutions?” The nurse explains that understanding what goes wrong in his social relationships might help prevent it from happening again. Hans mentions that he has heard of people taking medication to improve their mood and wonders if that could be his solution.

**Vignette 2: Inge**

Inge is 65 years old. She is a retired schoolteacher, recently celebrated her silver wedding anniversary with her husband, and has three adult children and three grandchildren. Inge has generally enjoyed good mental well-being throughout her life, but over the past few years, she has felt more down and lacking in energy.

During her annual chronic care consultation for her ischemic heart disease, the nurse tells her about a research project on mental well-being among patients with type 2 diabetes and ischemic heart disease. Inge finds it intriguing and wants to participate but has many questions. She asks, “Are the sessions really only half an hour—can that be enough?”

During the first session, Inge is given a book, and over the first two sessions, she and the nurse complete the first two steps in the book. Initially, Inge lists the problems she is currently facing. She speaks at length about her poor mental state and outlines various problems it causes for her.

In the next session, Inge is asked to select one problem to work on. She quickly decides to focus on how her lack of energy leads to self-isolation and feelings of loneliness. The rest of the session is spent discussing this issue.

During the third session, the nurse wants to move on to the next step in the book, which involves discussing possible solutions to Inge’s problem. However, Inge is somewhat skeptical. She doesn’t understand why they are not delving deeper into the problem and her feelings about it. Inge tells the nurse, “I feel like we haven’t really addressed the problem enough.”
